# The prevalence, awareness and control rate of hypertension among elderly in northwest of Iran

**DOI:** 10.15171/jcvtr.2016.35

**Published:** 2016-12-30

**Authors:** Samad Ghaffari, Leili Pourafkari, Arezou Tajlil, Mohammad Hassan Sahebihagh, Asghar Mohammadpoorasl, Jafar Sadegh Tabrizi, Nader D Nader, Akbar Azizi Zeinalhajlou

**Affiliations:** ^1^Cardiovascular Research Center, Tabriz University of Medical Sciences, Tabriz, Iran; ^2^University at Buffalo, Buffalo, New York, 14214, USA; ^3^Health Services Management Research Center, Tabriz University of Medical Sciences Tabriz, Iran

**Keywords:** Hypertension in Ederly, Epidemiology, Gender Differences

## Abstract

***Introduction:*** Adequate treatment of hypertension is infrequent in older patients. Determining the prevalence of hypertension in older patients, identifying the pattern of the treatment in this age group and evaluating their awareness of the disease may help healthcare systems to devise appropriate programs for controlling the disease.

***Methods:*** This descriptive cross sectional study included a sample from population of Tabriz, a large city in North-West of Iran, who were 60 years or older. Data collection and blood pressure measurements were conducted in the households of the participants from 1st June 2015 through 1st August 2015. Hypertension was defined as systolic and/or diastolic blood pressure (DBP) ≥150/90 mm Hg or receiving anti-hypertensive medications. Prevalence and determinants of hypertension, awareness of patients about their diagnosis and prevalence of treatment and adequately controlled blood pressure were determined.

**Results:** The prevalence of hypertension was 68.0%. Among hypertensive patients 81.8% were aware of their diagnosis, 78.0% were receiving antihypertensive medications. Among treated patients, 46.2% were adequately controlled. In univariate analysis, prevalence of hypertension was significantly higher in women (74.0% vs. 60.7%, *P* < 0.001). Women were more likely to be aware of diagnosis and to receive antihypertensive medications; however, the prevalence of adequately controlled blood pressure was similar in treated men and women. Among included variables in logistic regression analysis, older age, lower number of family members in household, cardiac diseases, being on low salt low fat diet, higher Body mass index (BMI) and not being educated were independently associated with having hypertension.

***Conclusion:*** Hypertension is highly prevalent among older population of Tabriz. Despite high rate of treatment, the rates of control are relatively low, indicating a demand for prevention and better management of hypertension in older individuals.

## Introduction

Hypertension increases the risk for developing various non-communicable diseases and is a major risk factor for myocardial infarction, stroke and chronic kidney disease. Along with that, hypertension imposes substantial financial burden on health care systems.^1^ However, hypertension is a preventable as well as a controllable disease, making it an important matter to consider when planning for public health policies.^2-4^

Although the global prevalence of hypertension is estimated to be about 22% in adult population of 18 years or higher, the prevalence differs between countries and among age groups.^5^ While the prevalence of hypertension is declining in higher income countries, lower income countries are encountering an increasing number of patients with hypertension.^5,6^ Since demographic and social changes in these societies contribute to this issue, improving lifestyle and increasing the awareness of patients about the disease can help healthcare systems overcome the problem.^5^ On the other hand, developing countries encounter a growing number of aging populations. Even though prevalence of hypertension increases with age,^7,8^ adequate treatment is infrequent in older patients.^2^ Consequently, identifying the pattern of disease prevalence and risk factors is essential for devising a targeted screening program and promoting the awareness of people about the modifiable risk factors of hypertension.

Regarding these facts, we devised a cross-sectional study focused only on older population to determine the prevalence of hypertension in Tabriz, the largest city in northwest of a middle-income country, Iran. The awareness of patients about their disease and prevalence of appropriate control of blood pressure and its determinant were also investigated in our study.

## Methods and Materials

### Study population

This descriptive cross sectional study included a sample from Tabriz population, who were 60 years or older. Tabriz is the largest city in North-West of Iran, a middle-income country located in Middle East. Data collection and BP measurements were conducted in the households of the participants from first June 2015 through first August 2015.

### Sample number and sampling methods

In accordance with the Cochran’s sample size formula (n=t^2^*p*q/d^2^) with 95% CI, *P *= Q and d=0.03, the sample size was calculated to be 1067 cases. Participants were selected by probability sampling and classified using multistage clustering and probability proportional to size (PPS) technique. The study comprises representative household surveys in Tabriz.

Respondents were selected using a multistage, stratified, random cluster sampling design with every individual having a known non-zero probability of being selected. The primary sampling units were stratified by Municipal districts. The sample size was chosen according to the population of elderly in each municipality proportional to the population of whole city, Population data of the municipality and city blocks were obtained from statistical center of Iran and PPS method was used for selecting relevant blocks. Finally, 10 elderly people were selected from each city block randomly. If such a person was not available, the next sample was replaced from the right on the basis of available addresses. Validity and reliability of the study was evaluated by pilot study on 45 people (five from each municipal districts).

### Study variables

To obtain demographic and health related information, trained health workers for interviewing older people, visited the participants in their homes. Interviewers filled out the prepared questionnaires. Demographic information included questions regarding age, city of born, marital status, number of children, number of household members, educational level and employment status. The participants were asked to provide subjective information about cardiovascular comorbidities including diabetes mellitus, smoking status, hyperlipidemia, history of coronary artery disease, and family history of hypertension. The information regarding the amount of physical activity and dietary habits, awareness about hypertension diagnosis and annual physician visits were also gathered. Medical history was gathered by asking from patients along with reviewing their prescriptions.

After volunteers rested for five minutes, trained interviewers measured their blood pressure from right and left arm, using sphygmomanometer and auscultatory method of measurement. While the arm of the patient was supported on the heart level, the bladder of appropriately sized blood pressure cuff was placed on midline over the brachial artery pulsation.

### Definition of variables

Hypertension was defined as systolic blood pressure of 150 mm Hg or more and/or diastolic blood pressure of 90 mm Hg or more in right and/or left arm. Using anti-hypertensive medications for treatment of raised blood pressure was also considered as having hypertension regardless of the level of blood pressure measured at the time of the study. Isolated systolic hypertension (ISH) was defined as systolic blood pressure (SBP) of 150 mm Hg or more and diastolic BP of less than 90 mmHg.

Participants who were on anti-hypertensive medications and their measured blood pressure was <150/90 mm Hg, were considered as the group with controlled blood pressure.

A person with hypertension was considered “aware” if he/she gave a positive response to the question, “Have you ever been told by a doctor or other health professional that you had hypertension, also called high blood pressure?” A person with hypertension was classiﬁed as “treated” if he/ she reported taking antihypertensive medication at the time of the survey. A treated person was considered “controlled” if his/her average SBP was <150 mm Hg and average diastolic blood pressure (DBP) was <90 mm Hg.

Diabetes mellitus was defined by a positive response to any of the questions, “Have you ever been told by a doctor that you have diabetes?”, “Are you now taking insulin?”; “Are you now taking diabetes pills to lower your blood sugar?”

Height was measured using a stadiometer and weight was measured using a weighing scale that was periodically calibrated. Body mass index (BMI) was calculated by dividing body weight in kilograms to square of height in meters.

### Study design

The prevalence of hypertension was determined in study sample. Patients with and those without hypertension were compared and independent predictors of hypertension were determined by multivariate logistic regression analysis. Further, patients under treatment with anti-hypertensive medications were allocated into two groups and compared regarding appropriate control of blood pressure. The independent predictors of having controlled blood pressure were determined. To evaluate sex related differences, demographic and clinical data were compared between men and women with hypertension.

### Statistical analysis

Data were analyzed using the statistical software SPSS-16 (Chicago, IL, USA). Categorical variables were expressed as frequencies and percentages and compared with chi-square test between groups. Continuous variables were described as mean ± standard deviation (SD) and compared between two groups using independent *t* test. To evaluate the independent predictors presence of hypertension in study population multivariate logistic regression analysis was performed. The inclusion of variables in the final regression model was based on likelihood ratio test. A probability value of less than 0.05 was considered as statistically significant.

## Results

### General characteristics

The mean age of patients was 70.1± 8.2 years and 514 (48.0%) of 1071 patients were male. The place of born was a rural area in 526 (49.1%) and an urban area in 514 (48.0%) patients. The mean number of family members living in one household was 3.1 ± 1.6 persons. The majority of individuals were married (764 cases, 71.0%) and 278 (26%), 15 (1.4%), 7 (0.7%), 7 (0.7%) were widowed, divorced, single, in other relationship status, respectively.

### Prevalence of hypertension and awareness of the disease

Among 1071 individuals included in our survey, 724 (67.6%) had hypertension. The measured systolic blood pressure was 150 mm Hg or more in 241 patients (22.5%). Diastolic blood pressure of 90 mm Hg or more was present in 235 (32.5%) of 724 patients. In 172 (23.8%) of 724 patients both SBP and DBP was above 150/90 mm Hg threshold.

Among 724 hypertensive patients, 585 (80.8%) were aware of their diagnosis and 565 (78%) were receiving antihypertensive medications but in just 261 patients (46.2%) out of 565 treated patients blood pressure was adequately controlled. The most common medication used by patients was beta-blockers, which was used by 306 (54.2%) of 565 patients, followed by angiotensin receptor blockers (ARBs) used by 281 patients (49.7%). Among patients under treatment with antihypertensive medications, 278 patients (49.2%) were using a single medication while 287 (50.8%) were using two antihypertensive medications or more.

Table 1 shows the differences between patients whose hypertension was adequately controlled and not adequately controlled with antihypertensive medications. As shown in the table, age, sex, marital status and history of comorbidities are not different between two groups. Likewise, being aware of their diagnosis, BMI, being on low sodium low fat diet and physical activity were not significantly different between two groups. However, patients who had regular annual ophthalmologic and cardiovascular check-up visits and those who had higher level of education were more likely to have controlled blood pressure. Monotherapy was also significantly associated with lower chance of having controlled hypertension (Table 1).

**Table 1:** Adequately controlled blood pressure in hypertensive patients under treatment with antihypertensive medications

		**Uncontrolled (n=304)**	**Controlled (n=261)**	**P value**
Age (y)		70.9± 8.0	70.4±7.9	0.518
Sex	Male	114 (37.5%)	109 (41.8%)	
	Female	190 (62.5%)	152 (58.2%)	0.344
Marital status	Single	2 (0.7%)	0 (0.0%)	
	Married	198 (65.1%)	183 (70.1%)	
	Divorced	7 (2.3%)	2 (0.8%)	
	Widowed	95 (31.3%)	74 (28.4%)	
	Other	2 (0.7%)	2 (0.8%)	0.316
Physical activity	None	91 (30.7%)	79 (30.4%)	
	15 Minutes a day	92 (31.1%)	68 (26.2%)	
	30 Minutes a day	80 (27.0%)	64 (24.6%)	
	60 Minutes or More	33 (11.1%)	49 (18.8%)	0.070
Diabetes mellitus		88 (28.9%)	78 (29.9%)	0.853
Cardiac disease		92 (30.3%)	100 (38.3%)	0.504
Asthma		21 (6.9%)	13 (5.0%)	0.434
Stroke		17 (5.6%)	14 (5.4%)	0.906
Anxiety disorders		36 (11.8%)	39 (14.9%)	0.432
Depression		36 (11.8%)	34 (13.0%)	0.776
Hyperlipidemia		24 (7.9%)	15 (5.7%)	0.402
Active smoking		24 (7.9%)	24 (9.2%)	
History of smoking		24 (7.9%)	31 (11.9%)	0.223
On low salt low fat diet		183 (65.1%)	179 (72.5%)	0.085
Annuals ophthalmologic and cardiovascular examinations		181 (65.1%)	181 (74.5%)	**0.026**
Dizziness		154 (52.9%)	127 (49.0%)	0.410
BMI classification	Underweight	44 (14.9%)	40 (16.4%)	
	Healthy Weight	122 (41.4%)	109 (44.7%)	
	Overweight	129 (43.7%)	95 (38.9%)	0.530
Literacy level	Illiterate	192 (63.2%)	139 (53.3%)	
	Primary	79 (26.0%)	62 (23.8%)	
	Secondary	17 (5.6%)	38 (14.6%)	
	Higher education	16 (5.3%)	22 (8.4%)	**0.001**
Number of family members in household	1	48 (15.8%)	23 (8.8%)	
	2	95 (31.3%)	98 (37.5%)	
	3	135 (44.4%)	117 (44.8%)	
	4	26 (8.6%)	23 (8.8%)	0.069
Awareness		291 (96.05)	257 (97.1%)	0.125
Monotherapy		167 (54.9%)	111 (42.5%)	**0.004**

### Comparison of individuals with and without hypertension

Table 2 shows the differences between patients with and those without hypertension. Patients who fulfilled the criteria of having hypertension were older than non-hypertensive individuals. Hypertensive individuals were significantly more likely to be female. Among 724 patients with hypertension, 412 (56.9%) were female but among 347 non-hypertensive individuals, 145 (41.7%) were female (*P* < 0.001). Hypertensive individuals were more likely to be illiterate, to have sedentary lifestyle and to be overweight. Diabetes mellitus and cardiac diseases were more prevalent in hypertensive group. Patients in hypertensive group were more aware of their blood pressure level and they were more likely to have annual ophthalmological and cardiovascular examinations and consume low-sodium low-fat diet. Waist circumference and waist to hip ratio were both higher in hypertensive group. Patients in non-hypertensive group were more likely to be active smoker and to have diagnosed cancer. The prevalence of asthma, hyperlipidemia, anxiety disorders and depression was similar in two groups (Table 2).

**Table 2:** Comparison of patients with and those without hypertension

		**Non Hypertensive (n=374)**	**Hypertensive (n=724)**	***P***
Age (y)		69.4±8.6	70.5±7.9	0.033
Sex	Male	202 (58.2%)	312 (43.1%)	
	Female	145 (41.8%)	412 (56.9%)	<0.001
The place of birth	Rural Region	162 (47.8%)	364 (51.9%)	
	Urban Region	177 (52.2%)	337 (48.1%)	0.236
Diabetes mellitus		52 (15.0%)	188 (26.0%)	<0.001
Cardiac diseases		58 (16.7%)	211 (29.1%)	<0.001
Asthma		13 (3.7%)	35 (4.8%)	0.517
Stroke		6 (1.7%)	31 (4.3%)	0.050
Anxiety disorders		35 (10.1%)	93 (12.8%)	0.229
Depression		29 (8.4%)	89 (12.3%)	0.089
Cancer		8 (2.3%)	3 (0.4%)	0.007
Hyperlipidemia		13 (3.7%)	42 (5.8%)	0.201
Active smoking	Never	256 (74.0%)	579 (80.4%)	
	Active	54 (15.6%)	71 (9.9%)	
	Quitted	36 (10.4%)	70 (9.7%)	0.019
Passive smoker		41 (12.2%)	96 (13.7%)	0.589
Medications for diabetes		38 (11.0%)	173 (23.9%)	<0.001
Oral Hypoglycemic agents		34 (9.8%)	154 (21.3%)	<0.001
Insulin		7 (2.0%)	24 (3.3%)	0.303
Aware of blood pressure		189 (55.3%)	531 (73.8%)	<0.001
Having medical insurance		320 (92.5%)	663 (91.7%)	0.748
Having complimentary health insurance		182 (53.5%)	347 (50.2%)	0.321
Double glazed windows in house		26 (7.5%)	35 (4.9%)	0.109
Marital status	Married	268 (77.2%)	496 (68.5%)	0.003
Physical activity	None	71 (20.8%)	197 (27.6%)	
	15-30 minutes	80 (23.4%)	213 (29.9%)	
	30-60 minutes	94 (27.5%)	186 (26.1%)	
	60 minutes or more	97 (28.4%)	117 (16.4%)	<0.001
On low fat low salt diet		161 (50.0%)	423 (62.8%)	<0.001
Annual ophthalmologic and cardiac examinations		155 (49.1%)	412 (61.6%)	<0.001
Chest pain		69 (20.2%)	247 (34.8%)	<0.001
Dizziness		122 (36.4%)	340 (48.2%)	<0.001
Being head of family		219 (64.2%)	421 (58.6%)	0.195
BMI classification	Underweight	94 (29.1%)	110 (16.1%)	
	Healthy Weight	150 (46.4%)	309 (45.2%)	
	Overweight	79 (24.5%)	264 (38.7%)	<0.001
Waist circumference (cm)		96.8±15.2	103.0±13.0	<0.001
Waist to hip ratio		0.95± 0.09	0.97±0.07	0.024
Educational level	Illiterate	154 (44.4%)	431 (59.6%)	
	Primary	111 (32.0%)	176 (24.3%)	
	Secondary	56 (16.1%)	74 (10.2%)	
	Higher Education	26 (7.5%)	42 (5.8%)	<0.001

### Independent predictors of hypertension in patients ≥ 60 years

Among included variables in logistic regression analysis, older age, lower number of family members in household, cardiac diseases, annual cardiovascular and ophthalmologic exam, being on low salt low fat diet, higher BMI and not being educated were independently associated with having hypertension (Table 3). However, sex, active smoking, diabetes, being married and having more than 30 minutes of daily physical activity were not independently associated with having hypertension (Table 3)

**Table 3:** Independent predictors of having hypertension in multivariate logistic regression analysis

	**Odds Ratio**	**95% CI**	**P value**
Age	1.027	(1.004-1.051)	0.021
Male sex	0.888	(0.599-1.317)	0.556
Number of family members (Continuous)	0.903	(0.820-0.994)	0.037
Diabetes	0.719	(0.482-1.072)	0.106
Cardiac disease	1.545	(1.050-2.275)	0.027
Low fat low salt diet	1.469	(1.066-2.025)	0.019
Annual cardiovascular and ophthalmologic exam	1.578	(1.141-2.182)	0.006
Body mass index (continuous)	1.096	(1.060-1.134)	<0.001
Being married	0.744	(0.490-1.128)	0.164
Active smoking	0.959	(0.605-1.519)	0.858
Activity more than 30 minutes a day	0.819	(0.585-1.146)	0.244
Educated	0.602	(0.428-0.846)	0.004

### Comparison of hypertensive males and females

Table 4 illustrate the differences between men and women with hypertension. Males were older than females (females: 69.6±7.7 years vs. males: 71.7±8.1 years, *P *< 0.001) with higher level of education. Males were more likely to be married and to live in a nuclear family. Compared to men, women were significantly more aware of their diagnosis (women: 87.8% vs. men: 77.9%, *P *= 0.001). Females were more likely to be under treatment with antihypertensive medications. (83.0% vs. 71.5%, *P *< 0.001). The prevalence of adequately controlled blood pressure in treated individuals was similar in males and females (44.4% vs. 48.9%, *P *= 0.344). Females had higher BMI level and lower physical activity level in comparison to males. The prevalence of diabetes mellitus was higher in females than in males. Females were more likely to receive monotherapy for treatment of hypertension 42.2% vs. 33.3%, *P *< 0.001). Waist to hip ratio was higher in females but waist circumference was similar in two groups. The rate of having health insurance was similar in males and females (90.8% of females vs. 92.9% of males, *P *= 0.356) but males were more likely to have supplemental health insurance plans 44.5% of females vs. 57.6% of males, *P *= 0.001).

**Table 4:** Sex-related differences in hypertensive patients

	Male N=312	Female N=412	P value
Age (Years)		71.7± 8.1	69.6±7.7	<0.001
Place of Birth	Rural Area	156(52.0%)	208(51.9%)	
	Urban Area	144(48.0%)	193(48.1%)	0.973
Marital Status	Single	1(0.3%)	2(0.5%)	
	Married	279(89.4%)	217(52.7%)	
	Divorced	4(1.3%)	10(2.4%)	
	Widowed	24(7.7%)	181(43.9%)	
	Other	4(1.3%)	2(0.5%)	<0.001
Education	Illiterate	123(39.4%)	307(74.5%)	
	Primary	101(32.4%)	75(18.2%)	
	Secondary	51(16.3%)	23(5.6%)	
	Higher education	37(11.9%)	7(1.7%)	<0.001
Type of Family	Extended	181(58.0%)	229(55.6%)	
	Nuclear	115(36.9%)	98(23.8%)	
	Living Alone	15(4.8%)	81(19.7%)	
	Other	1(0.3%)	4(1.0%)	<0.001
Number of Family Members	1	15(4.8%)	79(19.2%)	
	2	114(36.5%)	130(31.6%)	
	3	164(52.6%)	161(39.1%)	
	4	19(6.1%)	42(10.2%)	<0.001
Aware of Blood Pressure level		225(72.1%)	306(75.0%)	0.432
Last Measurement of Blood pressure	Within a Week	128(42.2%)	176(44.9%)	
	Within a Month	83(27.4%)	119(30.4%)	
	Within Six Months	60(19.8%)	74(18.9%)	
	Within a Year	32(10.6%)	23(5.9%)	0.131
Low Sodium, low Fat Diet		186(63.7%)	237(62.0%)	0.718
Physical Activity	None	45(14.5%)	152(37.8%)	
	15-30 Minutes	76(24.4%)	137(34.1%)	
	30-60 Minutes	104(33.4%)	82(20.4%)	
	60 minutes or more	86(27.7%)	31(7.7%)	<0.001
Annual Ophthalmologic and Cardiovascular Examination		186(62.2%)	226(61.1%)	0.828
Aware of Hypertension Diagnosis		233(77.9%)	352(87.8%)	0.001
Head of Household		295(95.5%)	126(30.8%)	<0.001
Health Insurance		290(92.9%)	373(90.8%)	0.356
Supplemental Health Insurance Plans		174(57.6%)	173(44.5%)	0.001
Smoking	None	192(62.1%)	387(94.2%)	
	Active Smoking	57(18.4%)	14(3.4%)	
	Quitted	60(19.4%)	10(2.4%)	<0.001
Passive Smoker		35(11.7%)	61(15.1%)	0.243
Diabetes Mellitus		68(21.8%)	120(29.1%)	0.013
Cardiac Diseases		89(28.5%)	122(29.6%)	0.814
Asthma		8(2.6%)	27(6.6%)	0.021
Stroke		16(5.1%)	15(3.6%)	0.427
Anxiety Disorders		33(10.6%)	60(14.6%)	0.140
Depression		33(10.6%)	56(13.6%)	0.267
Cancer		2(0.6%)	1(0.2%)	0.809
Hyperlipidemia		14(4.5%)	28(6.8%)	0.248
Other Chronic Diseases		70(22.4%)	105(25.6%)	0.396
Family History of hypertension		84(100.0%)	128(100.0%)	
Body Mass Index Classification	Underweight	59(19.7%)	51(13.3%)	
	Healthy Weight	167(55.7%)	142(37.1%)	
	Overweight	74(24.7%)	190(49.6%)	<0.001
Waist Circumference		102.1±12.4	103.7±13.5	0.120
Waist to Hip Ratio		0.98±0.08	0.95±0.06	<0.001
Awareness		233(77.9%)	352(87.8%)	<0.001
Treatment with Anti-hypertensive medications	Untreated	89(28.5%)	70(17.0%)	
	Treated	223(71.5%)	342(83.0%)	<0.001
Adequately Controlled Blood Pressure		109(48.9%)	152(44.4%)	0.344
Mono Therapy		104(33.3%)	174(42.2%)	<0.001

## Discussion

As shown in this study, the prevalence of hypertension, as a major cardiovascular risk factor is 67.6% in our elderly population and as expected the prevalence increases with age. Unlike our study, most studies in Iran have included different age groups with no special focus on elderly.^9-14^ In a report from North-East of Iran, hypertension defined as blood pressure ≥140/90 mm Hg was present in 62% of individuals older than 60 years.^9^ In comparison, the prevalence of hypertension in elderly differs among low to middle income countries.^6,15^ Sought Africa is reported to have a very high prevalence of 78 %, while the prevalence of hypertension in older population is 59% in China and 32% in India. However, it should be noted that in all these studies, unlike our survey the threshold for defining high SBP is set to be 140 mm Hg.^6^

Although the rise of blood pressure as a result of aging is inevitable, due to greater risk of cardiovascular diseases in older adults, proper treatment of hypertension as a major cardiovascular risk factor is mandatory.^2,16^ In our survey, 78% of hypertensive patients were aware of their hypertension but only 52.8% were under treatment with anti-hypertensive medications. The awareness of diagnosis in our region is higher than previous reports from Iran. In a study from Northeast of Iran, only 42.6% of patients older than 60 years were aware of their diagnosis and 17.6% were under treatment with hypertensive medications.^9^ In a recent survey from south of Iran, only 22.1% of patients of 60-69 years and 11.6% of patients older than 70 years were aware of their diagnosis.^10^ In comparison to developed countries, the reported prevalence of awareness is generally lower in low to middle income countries. However, the Federation of Russia had a prevalence of 72%.^6^ The prevalence of awareness is higher in developed countries and is reported to be about 80% in United States.^17,18^ The increasing level of awareness in the United States as a result of national health programs and advocates for decreasing cardiovascular diseases^17^ underscores the importance and efficacy of such health policies to detect and treat the affected individuals.

Among patients who were receiving anti-hypertensive medications, blood pressure was adequately controlled in only 46% of patients. This number is consistent with the results of a report from central regions of Iran, Isfahan, in which the prevalence of uncontrolled blood pressure was about 60% in patients older than 65 years.^11^ In another report from southern parts of Iran including individuals of age 20-74 years, the prevalence of controlled blood pressure among treated patients of all age groups was 34%.^14^ In comparison, the rate of adequately controlled blood pressure in treated patients was 72.7% in a recent report from united states in which the blood pressure goal of <150/90 mm Hg was applied for defining at-goal blood pressure.^19^ Different underlying factors may contribute to successful treatment of hypertension in older patients. In our study, the higher level of education and annual visits for ophthalmologic and cardiovascular examinations were associated with better control of blood pressure. This may originate from better adherence and optimal prescription of medications in annual visits. Also, patients who were receiving a single antihypertensive medication was more likely to have uncontrolled blood pressure. Even though older patients are prone to experience adverse effects of anti-hypertensive medications, effective treatment of blood pressure in older ages is generally achieved with multi-pharmacy.^20,21^ However, physicians may tend to underuse anti-hypertensive medications to avoid side effects of medications and decrease the risk of hypoperfusion in older patients.^19-22^ Despite the fact that we used a threshold of 150/90 mm Hg for defining controlled blood pressure, the prevalence of uncontrolled blood pressure in treated patients is still high. Increasing the quality of care with optimum use of anti-hypertensive medications and promoting life-style changes in patients may help in lowering the prevalence of uncontrolled blood pressure in our region. In a systematic review including data from Iranian population, it is reported that many patients consider hypertension as a consequence of daily stresses and quit using medications after relief from common symptoms such as headache and dizziness.^23^ Increasing the awareness of patients about the benefits of lowering blood pressure may also promote the rate of controlled blood pressure in our population.

Another important finding of our study is the gender-related differences in prevalence of hypertension and associated comorbidities in affected individuals. According to our results, women are more likely to suffer from hypertension in older age groups. In comparison to men with hypertension, women are more likely to have concurrent diabetes mellitus, which further increases the risk of cardiovascular diseases.^2^ One of the important modifiable gender-related differences in our study population is lower level of physical activity in women compared to men. Along with increasing physical activity levels, reducing the sitting time with similar activity levels can also help in lowering the risk of cardiovascular diseases.^24^ In addition, hypertensive women in our region are more likely to be overweight. Since higher BMI level is associated with increased risk of cardiovascular diseases,^2^ increasing physical activity along with improving diet may also help patients to lose body weight and achieve lower BMI level. On the other hand, in comparison to women, men in our region are more likely to be unaware of their diagnosis. They are also less likely to receive anti-hypertensive medications. However, among treated patients, there is no significant difference in prevalence of controlled blood pressure. Our results regarding the sex-differences in prevalence of awareness and treatment with anti-hypertensive medications are in agreement with the results of the surveys took place in other regions of Iran^9,10^ as well as other low-middle income countries^6^ and United States.^25^ Promoting the awareness about the diagnosis of hypertension and potential benefits of treatment is essential, particularly among men.

## Conclusion

Prevalence of hypertension in older population is high in our region. Despite the fact that, awareness of the patients about their diagnosis is high, the prevalence of adequately controlled blood pressure in treated patients is low.

## Ethical approval

This study was reviewed and approved by deputy of research ethics committee of Tabriz University of Medical Science. Informed consent was obtained from all participants.

## Competing interests

Authors declare no conflict of interest in this study.
